# Distribution and species composition of potato viruses in the Novosibirsk region

**DOI:** 10.18699/vjgb-24-61

**Published:** 2024-09

**Authors:** V.S. Maslennikova, M.B. Pykhtina, K.A. Tabanyukhov, E.V. Shelikhova, K.I. Mosalev, A.V. Katokhin, A.A. Bondar, A.B. Beklemishev, M.I. Voevoda

**Affiliations:** Federal Research Center of Fundamental and Translational Medicine, Novosibirsk, Russia Novosibirsk State Agrarian University, Novosibirsk, Russia; Federal Research Center of Fundamental and Translational Medicine, Novosibirsk, Russia; Federal Research Center of Fundamental and Translational Medicine, Novosibirsk, Russia Novosibirsk State Agrarian University, Novosibirsk, Russia; Federal Research Center of Fundamental and Translational Medicine, Novosibirsk, Russia Novosibirsk State Agrarian University, Novosibirsk, Russia; Federal Research Center of Fundamental and Translational Medicine, Novosibirsk, Russia; Federal Research Center of Fundamental and Translational Medicine, Novosibirsk, Russia; Institute of Chemical Biology and Fundamental Medicine of the Siberian Branch of the Russian Academy of Sciences, Novosibirsk, Russia; Federal Research Center of Fundamental and Translational Medicine, Novosibirsk, Russia; Federal Research Center of Fundamental and Translational Medicine, Novosibirsk, Russia

**Keywords:** Solanum tuberosum, viral infections, RT-PCR, potato Y virus, phylogenetic analysis, Solanum tuberosum, вирусные инфекции, ОТ-ПЦР, Y-вирус картофеля, филогенетический анализ

## Abstract

Among the many diseases that affect potato plants, viral infections are the most common and cause significant damage to farms, affecting both the yield and quality of potatoes. In this regard, an important condition for preserving the potato seed fund in Russia is systematic monitoring and early highly specific detection of potato viral infections. The purpose of the work is to study samples of potato varieties collected in the Novosibirsk region for the presence of viral infections using RT-PCR. 130 potato plants from three districts of the Novosibirsk region (NR) were studied. As a result of monitoring, the following viruses were identified: PVY (potato virus Y), PVS (potato virus S), PVM (potato virus M) and PVX (potato virus X). The quarantine pathogen potato spindle tuber viroid (PSTVd) was not detected in any of the samples analyzed. The maximum frequency of occurrence in the region was noted for three viruses: PVY, PVM and PVS. A significant proportion of the samples were mixed viral infections: the occurrence of the combination of infection PVY + PVM in plants was 25.0 %, and PVY + PVS, 22.6 %. To develop methods for determining the strain affiliation of the studied samples, the nucleotide sequences of the capsid protein genes of 10 Y-virus isolates were sequenced. Phylogenetic analysis of the studied sequences of NR isolates was carried out with a set of sequences of reference strains 261-4, Eu-N, N:O, NE-11, NTNa, NTNb, N-Wi, O, O5, SYR_I, SYR_II and SYR_III retrieved from GenBank. As a result of phylogenetic analysis, it was established that NR viral samples fell into two groups of strains: group 1, which also includes isolates of the reference strains 261-4/SYR_III, and group 2, NTNa. The obtained results of the strain affiliation of NR samples lay the basis for the development of DNA and immunodiagnostic systems for identifying PVY circulating in NR, as well as for elucidating the source and routes of entry of specific virus strains.Key words: Solanum tuberosum; viral infections; RT-PCR; potato Y virus; phylogenetic analysis.

## Abstract (ru)

Среди множества болезней, поражающих растения картофеля, именно вирусные инфекции являются наиболее распространенными и наносят значительный ущерб хозяйствам, влияя как на урожайность, так и на качество картофеля. В связи с этим важное условие сохранения семенного фонда картофеля в России – систематический мониторинг и раннее высокоспецифичное обнаружение вирусных инфекций картофеля. Целью работы было исследование образцов сортов картофеля, собранных на территории Новосибирской области (НСО), на наличие вирусных инфекций методом ОТ-ПЦР. Изучено 130 растений картофеля из четырех районов Новосибирской области. В результате мониторинга обнаружены следующие вирусы: PVY (potato virus Y), PVS (potato virus S), PVM (potato virus M) и PVХ (potato virus X). Ни в одном из анализируемых образцов не найден карантинный объект – вироид веретеновидности клубней картофеля (potato spindle tuber viroid – PSTVd). Максимальная частота встречаемости в районах области была отмечена для трех вирусов – PVY, PVM и PVS. Смешанные вирусные инфекции составили заметную долю образцов: встречаемость комбинации инфекции PVY + PVM в растениях составляла 25.0 %, PVY + PVS – 22.6 %. Для отработки методов выяснения штаммовой принадлежности изучаемых образцов проведено секвенирование нуклеотидных последовательностей капсидных белков 10 изолятов Y-вируса. С просеквенированными последовательностями был осуществлен филогенетический анализ совместно с набором последовательностей референсных штаммов 261-4, Eu-N, N:O, NE-11, NTNa, NTNb, N-Wi, O, O5, SYR_I, SYR_II, SYR_III, взятых в GenBank. В результате филогенетического анализа установлено, что образцы из НСО распределились в две группы штаммов: группа 1, включающая также изоляты референсных штаммов 261-4/SYR_III, и группа 2 – NTNa. Полученные результаты штаммовой принадлежности образцов из НСО закладывают основу для разработки ДНК- и иммуннодиагностических систем для выявления PVY, циркулирующих в НСО, а также для выяснения источника и путей проникновения конкретных штаммов вируса

## Introduction

The Novosibirsk region is a favorable region for potato growing (Batov, Gureeva, 2023). The area of its cultivation in the industrial potato growing sector (data on agricultural organizations and peasant farms, excluding households) of the Novosibirsk region in 2023 amounted to 3.8 thousand hectares, which is 6.2 % (0.2 thousand hectares) more than in 2022. At the same time, the total potato harvest in the industrial potato growing sector of the Novosibirsk region amounted to 74.9 thousand tons, which is 12.9 % (8.5 thousand tons) more than in 2022. The top 10 districts in the Novosibirsk region by the size of the harvested potato area in 2023 included: Novosibirsk (36.8 % of the total area), Ordynsky (25.6 %), Moshkovsky (18.6 %), Karasuksky (5.2 %), Toguchinsky (4.4 %), Cherepanovsky (3.3 %), Suzunsky (1.8 %), Iskitimsky (1.7 %), Kochenevsky (1.3 %), Bagansky (0.4 %). The remaining districts accounted for a total of 1.0 % (https:// ab-centre.ru/news/rynok-kartofelya-novosibirskoy-oblasti ---klyuchevye-tendencii).

According to the Federal State Statistics Service, the average potato yield in Russia is about 16 t/ha (https://rosstat. gov.ru/enterprise_economy), in the Novosibirsk region it is 22.5 t/ ha (Batov, Gureeva, 2023), while the maximum productivity of individual varieties of this crop can reach 400 t/ ha (State Register of Selection Achievements…, https://gossortrf. ru/). Decrease in yield mostly depends on the influence of various external factors, including the prevalence of a large number of viral pathogens.

Currently, 40 phytopathogenic potato viruses have been identified in different countries and regions (Hameed et al., 2014; Onditi et al., 2021). The most important of them, which have become ubiquitous wherever potatoes are grown, are potato leaf roll virus (PLRV), potato virus Y (PVY), potato virus X (PVX), potato virus S (PVS), potato virus M (PVM). Each of these pathogens is capable of causing yield losses of 10 to 60 %, and, in case of mixed virus infection, losses can be even higher (Byarugaba et al., 2020).

PVY is the fifth most important plant virus in the world (Scholthof et al., 2011) and causes the greatest economic losses in potato production, but also affects other common crops such as tomato, pepper, and tobacco (Kerlan, Moury, 2008; Lacomme et al., 2017). The PVY genome is highly variable and is susceptible to recombination. PVY exists as a complex of strains that can be defined based on hypersensitivity reactions (HR) to three known potato N genes (Jones, 1990; Chikh-Ali et al., 2014) or based on genome sequences and recombination patterns (Karasev, Gray, 2013; Green et al., 2017). Currently, fourteen PVY strains have been identified (Karasev, Gray, 2013; Green et al., 2017), including five nonrecombinants (PVYO, PVYEu-N, PVYNA-N, PVYC, and PVYO-O5) and nine recombinants (PVY-N:O, PVY-N-Wi, PVY-NTNa, PVY-NTNb, PVY-NE11, PVY-E, PVY-SYR-I, -II, and -III) (Chikh-Ali et al., 2016a, b; Green et al., 2017). Fourteen additional recombinants and genome variants have also been reported (Green et al., 2018).

Since diseases caused by potato viruses are incurable in field conditions, early detection of these pathogens and determination of their species composition is an actual task for agriculture and is included in the subprogram “Development of potato breeding and seed production in the Russian Federation” of the Federal Scientific and Technical Program for the Development of Agriculture for 2017–2025.

Currently, there are three main methods for diagnosing the virus in potato tubers: real-time RT-PCR, enzyme-linked immunosorbent assay (ELISA), and immunochromatographic assay.

Previously, studies of virus load on potato agrocenoses were conducted in some regions of the Russian Federation. In 2016, in the Astrakhan region, a high incidence of the Y virus was recorded on all plantings of early reproductive potatoes, with the exception of the Krona variety, especially on the Impala (65–95 %), Red Scarlett (85 %) and Courage (60 %) varieties. In 2017, on the Impala variety, while a high incidence of PVY was maintained (60 % of plants), significant damage (50 % of plants) by PVS and PVM was observed (Fominykh et al., 2017). The frequency of PVS and PVM in the Republic of Bashkortostan was 87 % and 78 %, respectively, PVX – 12 %, PVY – 28 %. Up to 61.6 % of tubers were infected with two viruses (PVS+PVY, PVS+PVХ and PVM+PVY) and 2.8 % of samples were infected with a combination of three viruses. Only 6.9 % of the studied samples were virus-free (Khairullin et al., 2021).

Given the high incidence of viral infections in potato plants in various regions of Russia, early and accurate diagnostics of viral infections as well as study of the genetic polymorphism of individual strains of the most common virus species are extremely important. After the introduction of PCR diagnostic methods, abundant data on the genetic diversity of PVY strains began to appear, and it became possible to conduct more detailed studies aimed at identifying the sources and routes of spread of potato viruses. For example, based on the results of monitoring the occurrence of viruses in samples of 4 potato varieties (Red Scarlett, Silvana, Labella, Nevsky) using the RT-PCR method, it was found that 100 % of plants were infected with the X virus and 26.3 % were infected with the Y virus (Grigoryan, Tkachenko, 2019), and the infection of potatoes with the Y virus in the Perm’ region in 2019 was 100 % (Pechenkina, Boronnikova, 2020).

The studies by А.М. Malko et al. (2017) showed a high incidence of PVY in the Samara, Tver’, and Leningrad regions (33.3, 29.2, and 25.7 %, respectively), that of PVS in the Samara and Irkutsk regions (66.7 and 30.5 %, respectively), and that of PVM in the Tver’, Samara, and Nizhny Novgorod regions (25.0, 22.2, and 19.4 %, respectively) (Malko et al., 2017). Diagnostics of potato viral diseases using real-time PCR, conducted in 2019 in the Saratov region, detected PVY in two potato varieties, in the absence of visual plant lesions

Since 2015, the Federal Research Center for Potatoes named after A.G. Lorkh has been studying the serological and phytopathological characteristics of PVY isolates from various regions of the Russian Federation, including the Novosibirsk Region. Out of the seven identified isolates with PVY monoinfection in the material from the Novosibirsk Region, five isolates exhibited serological and phytopathological properties of PVYO/C (common strain and acropetal necrosis strain) (Uskov et al., 2022).

The aim of this work was to study the species composition of potato viruses of different varieties and categories and the incidence of plants in farms of the Novosibirsk region using molecular genetic methods to determine their prevalence in seed tubers, as well as to study the strain composition of individual PVY isolates.

## Materials and methods

The work was completed in 2023. The studies were conducted on 130 S. tuberosum potato plants from Iskitimsky (varieties Gala (RS1), Red Scarlett (E), Rosara (RS1)), Ordynsky (varieties Gala (RS1), Lady Claire (RS1), Rosara (RS1)), Kochenevsky (varieties Zlatka (SE), Rosara (RS1)) and Novosibirsk (varieties Gala (RS1), Red Scarlett (RS1)) districts of the Novosibirsk region (Table 1).

**Table 1.**
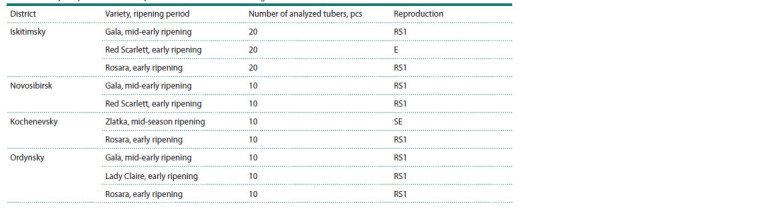
Analyzed potato material by districts of the Novosibirsk region

The samples were supplied by farms from the specified regions under an agreement with the Federal State Budgetary Institution “Rosselkhozcentr” in the Novosibirsk Region, which were selected in accordance with GOST 33996-2016. Ten samples were analyzed, the samples from the Iskitimsky district contained 20 tubers each, while the samples from the Ordynsky, Kochenevsky and Novosibirsk districts contained 10 tubers each. Potato tubers of each variety were cultivated in plastic pots (0.7 l) in boxes at a temperature of +24 °C ± 1 °C and a photoperiod of 16/8 hours: light/dark. Leaf samples for determining the viral load were collected four weeks after planting from the upper, middle and lower tiers of plants. Among the studied samples, four varieties (Rosara, Lady Claire, Gala, Red Scarlett) are varieties of foreign selection, and one variety (Zlatka) is of domestic selection. Isolation of viral RNA from the collected potato leaves was performed using the “PhytoSorb” kit manufactured by SYNTOL (Russia) in accordance with the manufacturer’s recommendations. RNA analysis was performed on a Rotor-Gene Q amplifier (Qiagen, Germany). The presence of viruses in potato leaf samples was determined using a reagent kit (by SYNTOL) PV-005 (PVX, PVY, PVM, PLRV, PVA, PVS and PSTVd).

Sample preparation for DNA sequencing. Individual Y-positive isolates (10 samples) were selected for cDNA synthesis and subsequent sequencing of the capsid protein gene region. Reverse transcription was performed using the RT M-MuLV-RH reagent kit (Biolabmix, Russia) according to the manufacturer’s protocol: 2–5 μg of total RNA was taken per reaction and primers (473-F: 5′-CAAATGACACAATCG ATGCA-3′; 474-R 5′-CATGTTCTTGACTCCAAGTAGA GTATG-3′) were designed for synthesis of the first and then the second strand of cDNA at the PVY genomic RNA site encoding the capsid protein of the virus. The primers were selected based on comparison of the nucleotide sequences of the envelope protein gene of known Y virus isolates represented in GenBank

The synthesized DNA was further used for PCR amplification of the coding region of the PVY capsid protein gene of the tested virus isolates. PCR was performed in a reaction mixture containing the above-mentioned primers 473-F and 474-R. The mixture was heated for 5 min at 70 °C and transferred to an ice bath for 2 min; then the mixture of the remaining reagents (RNA-dependent DNA polymerase, RT buffer, deoxynucleotide triphosphates) was incubated for 10 min at room temperature; then it was transferred to a thermostat at 42 °C for 2 h; at the end, the reaction was stopped by heating for 15 min at 70 °C. Quantitative PCR with real-time detection was performed using “BioMaster HS-qPCR SYBR Blue(2×)” by Biolabmix. PCR was performed in a CFX96 Touch amplifier (2014, Bio-Rad Laboratories, USA) according to the following amplification program: DNA denaturation at 95 °C for 1 min, followed by 40 PCR cycles (DNA denaturation at 95 °C for 20 s, primer annealing at 55 °C for 15 s, DNA chain elongation at 72 °C for 30 s). Amplification products were separated by gel electrophoresis in 0.8 % agarose gel containing 0.00005 % EtBr.

Sequencing of amplicons of the capsid protein gene of PVY isolates. The amplicons ~800 bp in size encoding the capsid protein of potato virus Y (PVY) were purified from PCR components of the reaction mixture by sorption on SpeedBead magnetic particles (GE Healthcare, USA) in the presence of 7 % PEG-8000. After washing three times with 80 % ethanol, amplicons were eluted with MiliQ water. For the Sanger sequencing reaction, 0.5 pmol of amplicon, 20 pmol of one of the primers (473_F_coat-Y-vir or 474_R_coat-Y-vir), 2 μl of BigDye v.3.1 reagent, 8 μl of 5X sequencing buffer (Nimagen, USA), 8 μl of 5M betaine and MiliQ water were used up to a total reaction volume of 40 μl. The temperature profile of the Sanger reaction consisted of: denaturation at 96 °C for 3 min, followed by 70 cycles (melting at 96 °C for 25 s; annealing at 40 °C for 10 s; elongation at 60 °C for 3 min) with a final warm-up at 98 °C for 5 min and storage until purification at 4 °C. The Sanger reactions were then purified from unreacted BigDye by gel filtration in tablet format microcolumns through Sephadex G-50 semisolid column (GE Healthcare, USA) by centrifugation at 1,700 g for 4 min. The products of the Sanger reaction were analyzed on an ABI 3500XL automated gene analyzer (Applied Biosystems, USA) at the Genomics CDC (ICBFM SB RAS). Nucleotide sequences of the studied amplicons were used for analysis by alignment and comparison with the GenBank database (NCBI, USA).

Comparison of nucleotide sequences of the covering capsid protein of the Y virus. For phylogenetic analysis, the nucleotide sequences of the capsid gene of PVY isolates from the Novosibirsk region were compared using the MAFFT service (https://www.ebi.ac.uk/Tools/msa/mafft/) with the corresponding reference sequences provided in GenBank (https://www.ncbi.nlm.nih.gov/genbank/). Analyses were per- formed with MEGAX software (Kumar et al., 2018) using maximum likelihood (ML) algorithm. Nucleotide sequencebased phylogram construction was performed considering all codon positions using evolutionary models of substitutions specified by the MEGAX>Models module: TN92(G+I) (Tamura–Nei). Phylogram construction based on amino acid sequences was performed using the JTT(G+I) module (Jones–Taylor–Thorton). The following named sequences were used as reference sequences for the cluster of strains: “261-4”: KY848023, AM113988, JF927755; “Eu-N”: KY847988, KY847986, JQ969036; “N:O”: KY847974, KY848018, AY884985, Z70238, AJ584851; “NE-11”: JQ971975, HQ912867; “NTNa”: AJ890344, M95491_i, AJ890345, AY884982; “NTNb”: AJ890343; “N-Wi”: KY847961, AJ890350, JQ924286, JN034046, AJ890349, KY847996; “O”: HQ912865, FJ643479, EF026074, AJ585196, JX424837; “O5”: FJ643477, U09509, HM367076, HQ912909, KY848035; “SYR_I”: GQ200836; “SYR_II”: AJ889867; “SYR_III”: AB461454. The bootstrap method (500 iterations) was used to determine the stability of the dendrograms.

Statistics. Virus occurrence was assessed using the χ2 test with Yates’ correction.

## Results

The highest frequency of occurrence in the districts of Novosibirsk region was noted for three viruses – PVY, PVM and PVS (Table 2). PVY was found in all the studied districts and affected all potato varieties, unlike the M and S viruses. The distribution of viruses across the districts of the Novosibirsk region was uneven (Table 3).

**Table 2.**
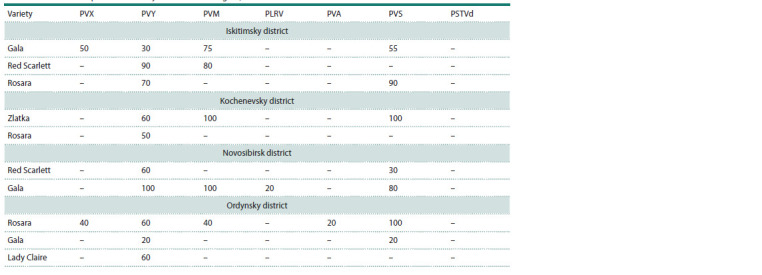
Prevalence of potato viruses by districts of the region, %

**Table 3.**
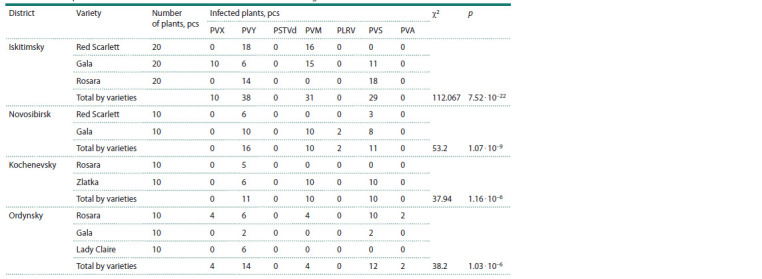
Cases of potato virus infection in different districts of the Novosibirsk Region Notе. The hypothesis about the prevalence of certain potato viruses in the districts of the region was tested using the χ2 criterion with Yates’ correction. P values are defined as р = 0.000.

The highest level of PVY infection was detected in the Novosibirsk district, where its prevalence on the Gala variety reached 100 %. Potato leafroll virus was detected on the same variety (20 %). PVS was found in all districts of the region, but the highest prevalence (30–100 %) was detected in the Ordynsky and Kochenevsky districts. Potato virus X was found in the Iskitimsky and Ordynsky districts (40–50 %). It should also be noted that due to the widespread cultivation of foreign varieties in our region, virus M was highly prevalent (40–100 %). Mid-early varieties (Gala, Zlatka) were more often affected by PVM than early-ripening ones. The highest viral load (PVX, PVY, PVM, PVA, PVS) was detected on the Rosara variety of the Ordynsky district. Potato spindle tuber viroid (quarantine object) was absent from all tested samples.

Mixed viral infections made up a significant proportion of the samples: the incidence of the PVY+PVM infection combination in plants was 25.0 %, PVY+PVS – 22.6 %, PVY+PVX – 3.8 % (Table 4). At the same time, the prevalence of “monoinfection” of any virus (PVS, PVM, PVX, PVY) was 19.4 %, and the number of plants in which there were no viruses was less than 1 %. Three viruses in the PVS+PVM+PVY combination were detected in 15.37 % of the samples, and four viruses were detected in 1.8 % (PVS+PVM+PVX+PVY).

**Table 4.**
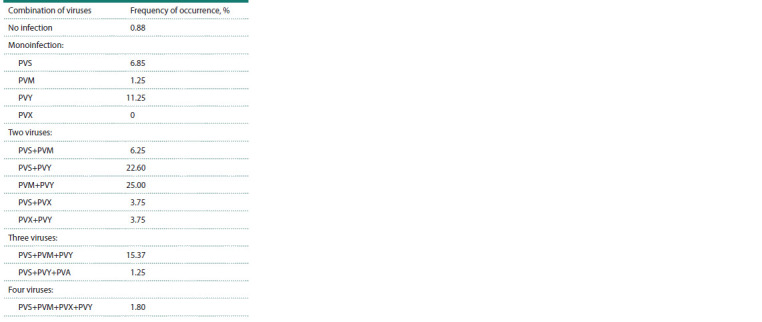
Frequency of occurrence of potato viruses

To determine the strain identification of the studied samples from the Novosibirsk region, amplified fragments of the PVY genome corresponding to the mature peptide of the capsid protein were sequenced and analyzed by phylogenetic methods using reference sequences from GenBank, described in detail in the article (Green et al., 2017, 2018). The registration numbers of the reference sequences are given in the “Materials and methods” section. The dendrograms obtained in the MEGAX program based on nucleotide and amino acid sequences made it possible to visualize the distribution of the reference strains used

The most compact group was formed by the strains of the O5 cluster, representing samples from North America with the eponymous serotype O5. This cluster was used as a proxy “outgroup” in constructing dendrograms to determine the approximate direction of evolution of PVY genetic diversity. The remaining clusters of strains were grouped less clearly. This can be explained by the fact that when constructing monolocus dendrograms, as in our case, there is no way to reflect the consequences of recombination events. Such events are known to occur all the time as viruses adapt to overcome the defenses of infected host plants and spread to new plants.

As is shown in the Figure, the samples from the Novosibirsk region were distributed into two groups of strains: group 1, including samples NSO01-05 and NSO08-09, is combined with the strains of the clusters “261-4” and “SYR_III”, and group 2, including samples NSO06-07 and NSO10, is combined with the strains of the cluster “NTNa”.

**Fig. 1.**
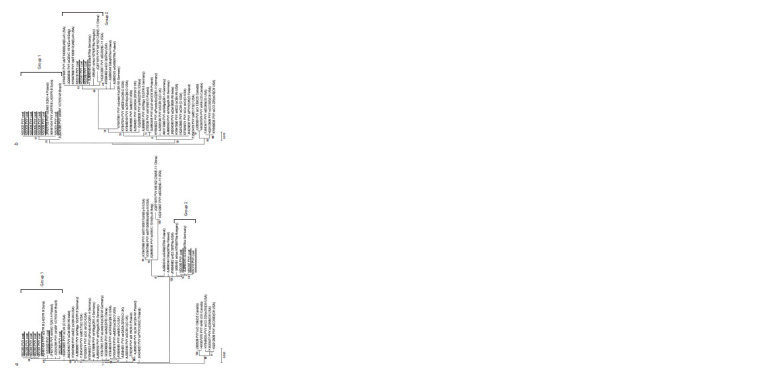
Phylogenetic analysis of PVY isolates from Novosibirsk region together with samples submitted to GenBank. Strain cluster designations and country of sample identification are indicated in parentheses. Serological classes are indicated for some samples, e. g. “sO” before the vertical dash. Samples from Novosibirsk region are underlined with a black dash. a – ML-dendrogram based on nucleotide sequences. b – ML-dendrogram based on amino acid sequences

Comparison of the topologies of the nucleotide and amino acid dendrograms also allows to make the expected conclusion that a significant part of the nucleotide diversity of viral sequences does not manifest itself at the level of encoded peptides. It is evident that the Novosibirsk region samples of the first group are identical to each other at the amino acid level and will probably have the same immunochemical properties in the case of using the epitopes of the mature capsid protein as a serological test. The same can be said about the Novosibirsk region samples of the second group. It can be expected that, in the presence of common epitopes, some of them will still differ so much between representatives of the two groups under study that it will be possible to develop differential serological tests.

## Discussion

Potato viral infections lead to a significant reduction in its yield, and therefore monitoring of the seed material contamination is a necessary measure for stable and sustainable production of this crop.

In this work, the RT-PCR method was used to monitor viral infections of seed potatoes in the Novosibirsk region, which revealed a high viral load. Among the analyzed samples, no differences in the distribution of viruses associated with varietal resistance and/or reproduction were found. Based on the analysis of the prevalence of viral infections, it was found that plants are most often infected with PVY, PVS and PVM viruses, which were found almost everywhere in the studied areas of the region with a frequency of 30–100 %. Unlike most other potato viruses, PVY is expanding its geographic distribution and causes economic damage to potato crops not only in Russia, but throughout the world (Byarugaba et al., 2020; Kreuze et al., 2020). Mixed viral infection including PVY is the most common (Kerlan, Moury, 2008), since most potato varieties are not resistant to it (Ahmadvand et al., 2012).

Potato plants grown in the Novosibirsk region were typically affected by two viruses (61.35 % of samples), of which PVM+PVY viruses were most common (25.0 %). The presence of three or four viruses simultaneously was detected in 16.62 % and 1.8 % of samples, respectively. Plants affected by viruses were stunted, leaf blades were underdeveloped. Rapid and premature growth of axillary buds was observed. Wrinkling and folding of leaves, their deep venation, chlorosis, and marginal necrosis were noted. This result confirms the results of other scientists (Khairullin et al., 2021), which showed that potatoes can be simultaneously infected with more than four viruses, including the most economically important viruses. The widespread distribution of viruses on potatoes is facilitated by the high infestation of fields with perennial weeds that act as reservoirs of viral infection (Szabó et al., 2020), and by the huge species diversity and the high number of carriers (Danci et al., 2009; Fox et al., 2017).

Since potato viral diseases are incurable, preventive measures aimed at using varieties resistant to viral infections and uninfected seed material are of great importance. These preventive measures require systematic early detection of viral infections, the absence of which has led to mass infection of potatoes with phytopathogens in Russia, including seed material. Therefore, the creation of highly sensitive, early and field-usable diagnostics of potato viral infections is an urgent task

PVY is considered one of the most significant viruses affecting both potatoes and other economically important species of nightshades (pepper, tomato, tobacco). Since, according to the results of our studies, the highest percentage of samples were infected with this type of virus, it was of interest to determine the nucleotide sequences of the capsid protein gene of the studied PVY isolates from the Novosibirsk region in order to determine the level of conservatism of these proteins for the subsequent creation of an immunochromatographic test system that is highly specific for the Siberian region. Phylogenetic analysis of the obtained samples revealed two groups of PVY strains among them: a group including the strains “261-4/SYR_III” and group 2 – “NTNa”. PVY is becoming increasingly widespread throughout the world, mainly due to the increase in the incidence of recombinant forms of the virus, such as PVYNWi and PVYNTN. These strains are highly virulent and have mild symptoms, which complicates their detection in seed potatoes.

Our data are consistent with the data of other authors who studied the strains of Y virus isolates in the territory of the Russian Federation. A.I. Uskov et al. (2016), when studying the strain composition of the Y virus of potato, common in the territory of the Russian Federation in 2015–2016, identified the ordinary strain PVYO in one variety sample, the tuber ring necrosis strain PVYNTN in 19, the recombinant strain PVYN:O in 36, and two strains PVYNTN and PVYN:O simultaneously in 53 variety samples. Based on a comparative analysis of the marker sequence of the 5′-untranslated region NTR locus, A.A. Stakheev et al. (2023) determined that potato virus Y isolates distributed in various territories of the Russian Federation belonged mainly to the necrotic and recombinant groups of strains, with the exception of a single isolate occupying an intermediate position between these two groups.

Determination of the PVY strain identification not only is of great importance in terms of improving strategies to combat this virus, but also has great diagnostic value. From a comparison of the topologies of the nucleotide and amino acid dendrograms, it follows that both groups of samples from the Novosibirsk region that we identified do not show intragroup differences at the amino acid level, which may indicate serological similarity of samples in a group and the prospects for developing differential serological diagnostics for samples from different groups.

## Conclusion

Thus, when developing DNA and immunodiagnostic systems for detecting PVY circulating in the Novosibirsk region, it is possible to use primarily the genetic variations of the virus of these strain clusters.

The obtained results of the strain identification of samples from the Novosibirsk region make the foundation for identifying the source and routes of penetration of specific strains of the virus, as well as for assessing the phytopathogenic risks for potato varieties used in the Novosibirsk region.

## Conflict of interest

The authors declare no conflict of interest.
